# GuREx-MIH: radiographic assessment of eruption patterns of second permanent molars and premolars in 11-year-olds after early extraction of the first permanent molar – a split-mouth trial

**DOI:** 10.1093/ejo/cjaf055

**Published:** 2025-08-14

**Authors:** Adnan Hajdarević, Christina Stervik, Nina Sabel, Birgitta Jälevik, Agneta Robertson, Ken Hansen, Emina Čirgić

**Affiliations:** Department of Pediatric Dentistry, Institute of Odontology, Sahlgrenska Academy, University of Gothenburg, SE-405 30, Gothenburg, Sweden; Folktandvården Björkekärr, Public Dental Service, Region Västra Götaland, SE-416 81, Gothenburg, Sweden; Department of Oral and Maxillofacial Radiology, Institute of Odontology, Sahlgrenska Academy, University of Gothenburg, SE-405 30, Gothenburg, Sweden; Department of Pediatric Dentistry, Institute of Odontology, Sahlgrenska Academy, University of Gothenburg, SE-405 30, Gothenburg, Sweden; Clinic of Pediatric Dentistry, Public Dental Service, Region Västra Götaland, SE-413 90, Gothenburg, Sweden; Department of Pediatric Dentistry, Institute of Odontology, Sahlgrenska Academy, University of Gothenburg, SE-405 30, Gothenburg, Sweden; Department of Pediatric Dentistry, Institute of Odontology, Sahlgrenska Academy, University of Gothenburg, SE-405 30, Gothenburg, Sweden; Clinic of Orthodontics, Public Dental Service, Region Västra Götaland, SE-413 90, Gothenburg, Sweden; Institute of Odontology, Sahlgrenska Academy, University of Gothenburg, SE-405 30, Gothenburg, Sweden; Department of Orthodontics, Folktandvården Stockholms län AB, Folktandvården Eastmaninstitutet, SE-113 24, Stockholm, Sweden

**Keywords:** molar-incisor hypomineralization, panoramic radiography, paediatric dentistry, tooth eruption, tooth extraction

## Abstract

**Background/Objectives:**

Molar-Incisor Hypomineralisation (MIH) affects 14% of the global population, often leading to compromised first permanent molars (FPM). Early extraction of severely affected FPMs may temporarily affect proper eruption and alignment of second permanent molars (SPM) and second premolars (SP). This study aimed to evaluate the eruption patterns of SPMs and SPs, and the overeruption of opposing FPMs, after early FPM extraction using panoramic radiographs in 11-year-old patients. A secondary aim was to assess radiographic quality for these evaluations.

**Subjects and Methods:**

This split-mouth trial included patients aged 6–9 with severe MIH requiring FPM extraction. Panoramic radiographs were taken pre-extraction (T0) and at age 11 (T1) to measure eruption length and angulation of SPMs and SPs. Radiographs were analysed using Facad software, and imaging errors were recorded. Paired t-tests compared extraction and non-extraction sides.

**Results:**

Among 47 patients, 31 had maxillary and 25 mandibular FPM extractions. At T0, eruption length and angulation of SPMs and SPs were similar between sides. At T1, maxillary SPMs erupted faster (13.5mm vs. 10.8mm, p < 0.001) and more upright (72.9° vs. 62.1°, p < 0.001) on the extraction side, while SPs showed increased mesial angulation (82.5° vs. 89.3°, p < 0.05). Mandibular SPMs and SPs showed no differences. No overeruption of opposing FPMs was observed. Measurement reliability was excellent (ICC: 0.997–0.999), despite 75 of 94 radiographic contained errors.

**Limitations:**

The three-year follow-up limits long-term insights, and radiographic distortions may affect reliability.

**Conclusions:**

Early FPM extraction impacts maxillary but not mandibular SPM and SP eruption patterns without causing overeruption of opposing FPMs by age 11. Radiographic techniques are essential to minimize incorrect patient positioning, as such factors may impact measurement reliability.

## Introduction

A condition of hypomineralised teeth is Molar-Incisor Hypomineralisation (MIH) which affects approximately 14% of the global population [1]. First permanent molars (FPM) impacted by MIH can present a significant burden for patients, causing hypersensitivity [2] and requiring frequent dental treatments due to the challenges associated with restorative procedures [3]. In cases with post-eruptive breakdown, the risk of rampant dental caries increases [4] and FPM may therefore need treatment shortly after eruption. As a result of extensive post-eruptive breakdown of hypomineralised enamel or dental caries, extraction may be the therapy of choice [5]. Early extraction of FPMs with poor prognosis is an accepted treatment choice [6–8], however, there are some scepticism within the profession considering extraction therapy of the FPM, while these teeth have a major role maintaining normal masticatory function [9].

Previous studies have reported a favourable outcome of mesial movement of the second permanent molar (SPM) after early extraction of compromised FPMs, with a higher success rate in the maxilla compared to the mandible [10–12]. Nevertheless, issues such as tipping and rotation of the SPM and second premolar (SP) may arise [11]. Thilander and Skagius identified the age of 8–10 years as an appropriate age for FPM extractions to facilitate spontaneous space closer through mesial movements of the SPMs [13]. In addition, a more recent study has demonstrated a high success rate in achieving spontaneous space closure when FPM extraction was performed during the SPMs Demirjian’s root development stages E and F [11]. A longitudinal retrospective study of adolescents concluded that extraction of FPMs affected with MIH, in combination with orthodontic space closure with fixed appliance, had favourable effect on the maxillary SPM and third molar angulation [14].

A panoramic radiograph provides an overview image that can be used for analysing teeth and surrounding structures. However, the panoramic technique is considered challenging, and studies have shown that obtaining an optimal image for diagnostic purposes can be difficult, potentially complicating accurate measurements [15]. Errors in radiographs, such as forward or backward tilting or rotations to the right or left of the head, can result in unreliable measurements of length and angulation of posterior teeth [16, 17]. Research has demonstrated that ratio calculations of vertical linear measurements and angulation on panoramic radiographs remain consistent and accurate when conducted on the same patient across different time points [18]. Despite these challenges, orthodontists often prefer panoramic radiographs for an initial overview of dental conditions, as it allows for a comprehensive assessment of the dentition and surrounding anatomy in a single image [19]. One significant advantage of panoramic imaging is its lower radiation dose compared to more advanced imaging techniques, such as cone-beam computed tomography (CBCT) or computed tomography (CT), making it a preferred choice in certain clinical settings [20–22].

Until now, no study has evaluated the eruption pattern of both maxillary and mandibular second permanent molars and second premolars following early extraction of the first permanent molars using panoramic radiographs. The purpose of this study was to evaluate panoramic radiographs to follow the eruption pattern of the second permanent molar and premolar, as well as the overeruption of the opposing first permanent molar, following the early extraction of the first permanent molar due to severe Molar-Incisor Hypomineralisation at 11 years of age. A secondary aim was to assess the quality of panoramic radiographs for determining the eruption pattern of the posterior teeth.

## Subjects and methods

The study was designed as a multicentre, split-mouth trial within the GuREx-MIH project (**G**othenburg **U**niversity, **R**estoration or **Ex**traction of first permanent molars due to severe **MIH**). This project evaluates various aspects of treatment, including restoration and extraction of first permanent molars (FPM), due to severe MIH [23].

Ethical approval was obtained from the Swedish Ethical Regional Board in Gothenburg, Sweden (Dnr: 352-15), in accordance with the World Medical Association Declaration of Helsinki. This trial was registered on ClinicalTrials.gov (registration number: NCT06228989).

### Sample size calculation

The sample size was determined using the alpha significance level of 0.05 and 80% power to detect a difference between the extraction and the control sides of 1.6 millimetres (SD 1.8) in the eruption length of the SPM [24]. Based on this calculation, at least 20 patients with unilateral extraction FPM were required for each jaw.

### Subject recruitment and eligibility criteria

Patients in the GuREx-MIH project were referred to the Clinics for Pediatric Dentistry at the Public Dental Service in Public Dental Service in Region Västra Götaland and Region Östergötland due to severe Molar-Incisor Hypomineralisation (MIH) of their first permanent molars (FPM). The inclusion criteria for this study were patients aged 6–9 years who had undergone unilateral extraction of FPMs due to severe MIH. Exclusion criteria encompassed dental agenesis in the analysed jaw or the presence of general disorders.

## Methods

A panoramic radiograph was taken before FPM extraction (T0) and at follow-up at age 11 (T1). At both T0 and T1, the following parameters were recorded: the presence of the third permanent molars, developmental stage of the second permanent molars (SPM) and second premolars (SP) using Demirjian stages (stage A-H) [25], and the eruption level of the persisting FPMs, SPMs, and SPs. Each patient served as their own control, comparing the extraction and the non-extraction sides.

Panoramic radiographs were analysed using Facad version 3.11.2.1616 (Ilexis AB, Linköping, Sweden). The software calibrated the radiographs based on their magnification ration, dots per inch (DPI; 25.4 mm).

**Figure 1.**
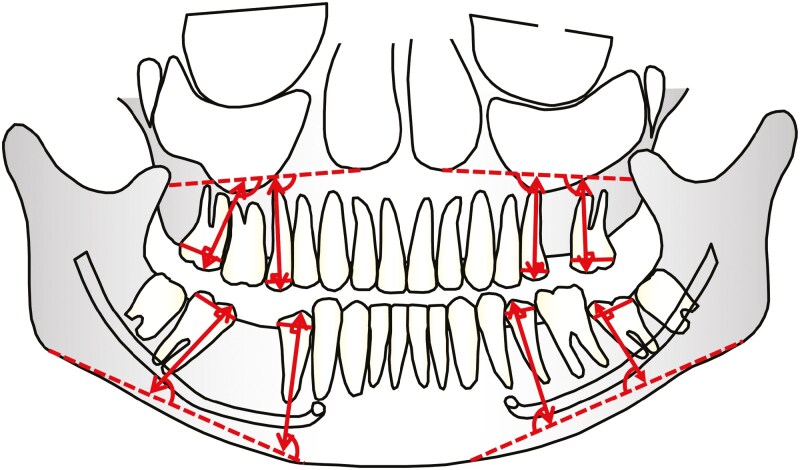
A schematic illustration of the panoramic radiograph, and the analysed measurement values: eruption length and angulation.

To calculate tooth angulation and analyse the eruption length, reference lines and points were identified on the panorama radiographs. In the maxilla, a reference line was drawn from the inferior margin of the zygomatic process, tangential to the lower border of the nasal cavity at the level of the lateral incisor root. In the mandibulae, a reference line was drawn as a tangential line from the lower border of the mandible, the reference line was drawn as tangential line from the lower border of the mandible, extending posteriorly to the SPM and anteriorly to the lower border at the level of the canine root. For each FPM, SPM and SP, the tip of the highest cusp, as well as the most mesial and distal points of the crown, were marked on the radiograph. A line between the mesial and distal points, representing the widest part of the tooth crown, was than constructed. (Fig. 1)

All patients were included in the analysis of the SP and SPM. However, for assessing the overeruption of the FPMs, only cases where the opposing FPM was extracted on one side were considered.

### Assessment of panoramic radiographs, and measurements

All panoramic radiographs were retrospectively evaluated for quality by two authors: a general dentist (A.H.) and a senior consultant in oral and maxillofacial radiology (C.S.), who reached a consensus on the number of errors. The radiographic assessment encompassed: (1) Upward rotation of the chin and occlusal plane, (2) Downward rotation of the chin and occlusal plane, (3) Anterior teeth widening, (4) Head rotation to the right, (5) Head rotation to the left, (6) Invisibility of the mandible’s lower border in the image, (7) Lack of tongue contact with the hard palate, and (8) Presence of foreign objects and/or other errors. Each error was recorded as either present or absent.

To estimate the method-error and assess reliability, examiner A.H. measured the eruption length on panoramic radiographs for ten randomly selected patients and repeated the measurements after two weeks to evaluate intra-examiner reliability. Additionally, inter-examiner reliability was assessed by having a second examiner, A.R., independently perform the same measurements on the same set of radiographs.

### Statistical analysis

All statistical analyses were performed using IBM SPSS Statistics version 29.0 (IBM Corp., Armonk, NY, USA). Descriptive statistics were calculated to assess inter-individual differences in tooth positions. The normality of continuous variables, including eruption length and angulation ratios for the SPM and SP, was assessed using the Shapiro–Wilk test. The data were found to be normally distributed (p > 0.05 for all variables), which supported the use of parametric tests.

Paired t-tests were conducted to compare the extraction and non-extraction sides at baseline (T0) and follow-up (T1). An independent t-test was used to compare the eruption rations between the maxillary and mandibular values. The Pearson correlation coefficient was used to explore associations between the position of the SPM and SP with root developmental stage (based on Demirjian stages) and the presence of third molars. Rooth development was assessed using the Demirjian method, which classifies root formation into stages A through H. For statistical analysis, these stages were converted to ordinal numeric values (A = 1, B = 2, … , H = 8) to facilitate correlation testing. This numerical ranking enabled the calculation of Pearson correlation coefficients between root developmental stage and eruption ratios (length and angulation) at follow-up. A significance level of p < 0.05 was applied for all statistical tests.

## Results

**Figure 2.**
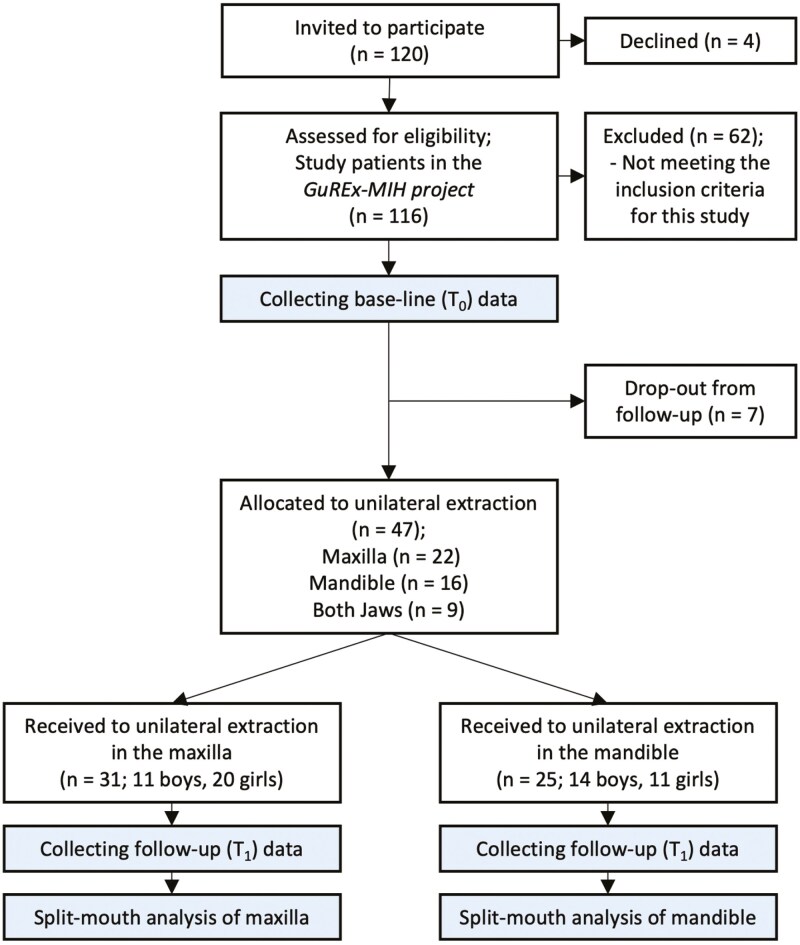
Flowchart for participants and drop-outs in the study.

This study included 47 patients, 20 boys and 27 girls, from the GuREx-MIH project. Among them, 31 had unilateral extractions in the maxilla, and 25 in the mandible. Fifteen patients had persisting antagonistic FPM and were eligible for overeruption analyses in both the maxilla and mandible. There were no significant age differences between maxilla and mandible groups at T0 or T1. The average follow-up period was 3.2 years (SD = 1.0) for the maxilla and 3.1 (SD = 1.0) years for the mandible. (Table 1; Fig. 2) At follow-up (T1), 3 out of 31 patients in the maxilla group and 1 out of 25 patients in the mandible group had fully erupted second permanent molars (SPM).

**Table 1.** Distribution of patients in the maxillary and mandibular groups.

Unilateral extraction of FPM	Maxilla		Mandible	
Patients, n	31		25	
Age in years, mean (SD)	8.1 (1.0)		8.2 (1.1)	
Extraction right side, n (%)	18 (58)		14 (56)	
Extraction left side, n (%)	13 (42)		11 (44)	
Demirjian stage of the SPM on the extraction side (T0 / T1)				
A B C D E F G H	- / - - / - 8 / - 20 / - 3 / 1 - / 6 - / 20 - / 4		- / - - / - 10 / - 10 / - 2 / 1 2 / 11 1 / 11 - / 2	
Demirjian stage of the SP on the extraction side (T0 / T1)				
A B C D E F G H	- / - - / - 5 / - 10 / - 15 / 1 1 / 7 - / 16 - / 7		- / - - / - 4 / - 11 / - 7 / 1 3 / 16 - / 5 - / 3	

FPM, first permanent molar; SPM, second permanent molar; SP, second premolar; n, number; %, percentage of patients; SD, standard deviation.

### Maxilla T0-T1

**Figure 3.**
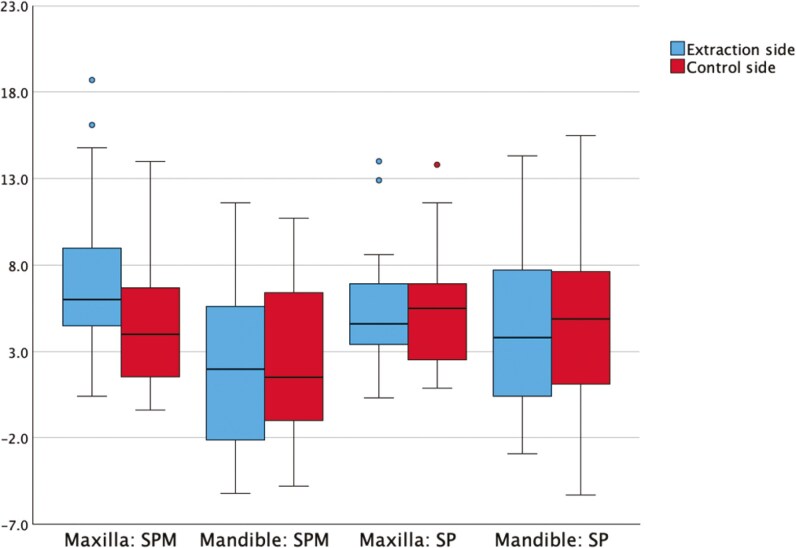
Bar chart of mean difference measurements of eruption length in mm of second permanent molar (SPM) and second premolar (SP).

**Figure 4.**
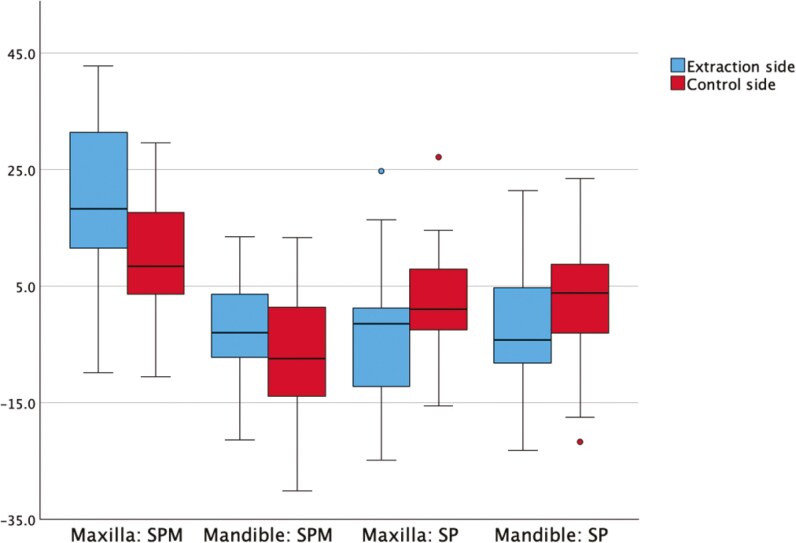
Bar chart of mean difference measurements of angulation in angulation degree of second permanent molar (SPM) and second premolar (SP).

At T0, the eruption length of the SPM and SP was similar between the extraction and the non-extraction side in the maxilla. By T1, the SPM had erupted more on the extraction side, while the SP show no difference in eruption length (Table 2; Fig. 3). Regarding angulation, no differences were observed at T0 between the extraction and non-extraction sides. At T1, the SPM on the extraction side was significantly more upright compared to the control side, whereas the SP had a larger mesial angulation on the extraction side (Table 3; Fig. 4).

**Table 2.** Mean ratio of eruption length and angulation, at baseline (T0) and follow-up at age of 11 years (T1), of the second permanent molar (SPM) and second premolar (SP).

	T0 (ratio: EX/NON-EX)		T1 (ratio: EX/NON-EX)		P-value
	mean	SD	mean	SD	
**Eruption length**					
Maxilla (n = 31)					
SPM	1.0	0.3	1.3	0.3	**<0.001**
SP	1.0	1.4	1.1	0.2	NS
Mandible (n = 25)					
SPM	1.0	0.0	1.0	0.1	NS
SP	1.0	0.1	1.0	1.0	NS
**Angulation**					
Maxilla (n = 31)					
SPM	1.0	0.1	1.2	0.1	**<0.001**
SP	1.0	0.2	0.9	1.2	**0.040**
Mandible (n = 25)					
SPM	1.0	0.1	1.0	0.1	NS
SP	1.0	0.2	1.0	0.1	NS

Values in bold represent statistically significant association (P ≤ 0.05; paired T-test). P-value, probability value; SD, standard deviation; NS, not significant.

**Table 3.** Pearson correlation coefficients (r) and p-values between the eruption parameters (length and angulation ratios at T1) of the second permanent molar (SPM) and second premolar (SP) and the developmental stage of the root and the presence of third molars, stratified by jaw. Root developmental stage was assessed according to Demirjian’s method (stages A–H) [25], and converted to ordinal numeric values (A = 1, B = 2, … , H = 8) for statistical analysis. The presence of third molars was recorded as a binary variable (1 = present, 0 = absent).

Ratio	Eruption length		Angulation	
Maxilla	SPM	SP	SPM	SP
Root developmental stage P-value	−0.247 NS	−0.067 NS	0.026 NS	−0.214 NS
Presence of third molar P-value	0.540 NS	N/A	0.087 NS	N/A
Mandible				
Root developmental P-value	0.074 NS	−0.227 NS	−0.106 NS	0.235 NS
Presence of third molar P-value	−0.151 NS	N/A	0.209 NS	N/A

P-value, probability value (P ≤ 0.05; Pearson correlation coefficient). NS, not significant; N/A, not applicable.

In the maxilla, no correlation was found between the ratio of eruption length and angulation of the SPM and the SP with the developmental stage of the root, or the presence of a third molar (Table 3). Additionally, no overeruption of the FPM was observed compared to the non-extraction side (Table 4).

**Table 4.** Mean ratio of the overeruption at baseline (T0) and follow- up at age of 11 years (T1), of the first permanent molar (FPM).

Overeruption length	T0 (ratio: EX/NON-EX)		T1 (ratio: EX/NON-EX)		P-value
	mean	SD	mean	SD	
Maxilla (n = 15)					
FPM	1.0	0.1	1.1	0.1	NS
Mandible (n = 15)					
FPM	1.0	0.1	1.0	0.0	NS

Values in bold represent statistically significant association (P ≤ 0.05; paired T-test). P-value, probability value; SD, standard deviation; NS, not significant.

### Mandible T0-T1

At T0, eruption length and angulation of the SPM and SP were similar between the extraction and non-extraction sides. By T1, no differences in eruption level or angulation were observed between the two sides. (Table 2; Fig. 3; Fig. 4)

In the mandible, no correlation was observed between the ratio of eruption length and angulation of the SPM and the SP, with the developmental stage of the root, or the presence of a third molar (Table 3). The FPM did not show overeruption on the side where the opposing FPM was extracted compared to the control side (Table 4).

### Eruption ratios between jaws

The comparison of eruption length and angulation ratios between the maxilla and mandible at T1 showed significant differences. The mean eruption length ratio of the SPM was significantly higher in the maxilla (1.31, SD 0.30) than in the mandible (1.01, SD 0.08; p < 0.001). Similarly, the angulation ratio of the SPM was significantly greater in the maxilla (1.17, SD 0.17) compared to the mandible (0.97, SD 0.09; p < 0.001).

In contrast, for the SP, the eruption length ratio did not differ significantly between maxilla (1.03, SD 0.20) and mandible (1.02, SD 0.11; p = 0.75). However, the angulation ratio of the SP was significantly lower in the maxilla (0.94, SD 0.22) than in the mandible (1.04, SD 0.13; p = 0.017).

### Assessment of panoramic radiographs, and measurements

Out of the 94 panoramic radiographs evaluated, 75 contained errors. The assessment results are presented in Table 5. The number of errors per radiograph of the evaluated panoramic radiographs are shown in Table 5. The number of errors per radiograph ranged from 0 to 5, with median number of 3 errors.

**Table 5.** Summarizing the distribution of different errors in panoramic radiographs at baseline (T0) and follow-up (T1).

	n	%
**Errors at T0;** Number of evaluated radiographs	47	100
Upward rotation of the chin and occlusal plane	26	55
Downward rotation of the chin and occlusal plane	4	9
Anterior teeth widening	26	55
Head rotation to the right	9	19
Head rotation to the left	7	15
Invisibility of the mandible’s lower border in the image	4	8
Lack of tongue contact with the hard palate	41	87
Presence of foreign objects and/or other errors	1	2
**Errors at T1;** Number of evaluated radiographs	47	100
Upward rotation of the chin and occlusal plane	19	10
Downward rotation of the chin and occlusal plane	7	15
Anterior teeth widening	13	28
Head rotation to the right	15	32
Head rotation to the left	5	11
Invisibility of the mandible’s lower border in the image	0	0
Lack of tongue contact with the hard palate	42	89
Presence of foreign objects and/or other errors	0	0

n, number; %, percentage of radiographs.

The Intraclass Correlation Coefficient (ICC) for intra-examiner reliability was 0.999 for eruption length (95% CI: 0.998–0.999) and 0.997 for angulation (95% CI: 0.996–0.998), indicating excellent agreement. For inter-examiner reliability, the ICC was 0.850 for eruption length (95% CI: 0.720–0.930) and 0.895 for angulation (95% CI: 0.810–0.950), indicating good agreement. All ICC values were calculated using a two-way mixed model with consistency type.

## Discussion

The present study found a significant difference in the angulation of the second permanent molar (SPM) and second premolar (SP), as well as eruption length of the SPM following early extraction of the first permanent molar in the maxilla. However, no correlation was observed between the ratio of eruption length and angulation of the SPM and SP with either the developmental stage of the root or the presence of a third molar.

In the maxilla, the study revealed that the SPM erupted significantly faster eruption on the extraction side compared to the non-extraction side at follow-up (T1). The accelerated eruption may be due to the absence of resistance from extracted FPM, allowing for unrestricted vertical growth.

Additionally, the SPM was found to be in a more upright position on the extraction side at T1, which is consistent with the findings of earlier studies indicating that the absence of the FPM may reduce mesial tipping tendencies in the SPM [26]. However, the SP in the maxilla showed greater mesial angulation on the extraction side at T1, possibly as a compensatory response to the increased space created by FPM extraction.

In the mandibular arch, no significant differences were observed in eruption length or angulation of the SPM and SP between the extraction and non-extraction sides at both T0 and T1. This suggests that the mandibular SPM and SP are less affected by FPM extraction compared to the maxillary teeth. These findings are consistent with previous longitudinal data, which indicate a differential eruption pace between the upper and lower jaws, with maxillary SPMs and SPs exhibiting a more pronounced and faster eruption pattern compared to their mandibular counterparts [24].

The study found no correlation between the eruption length or angulation of the SPM and SP and either the developmental stage of the root or the presence of a third molar. This suggests that while early FPM extraction can influence the vertical eruption of adjacent teeth, root development stage and third molar presence do not significantly influence how much the SPM and SP erupt or change in angulation.

A systematic review suggests that the presence of the lower third molar may increase the likelihood of space closure, but this finding remains uncertain due to methodological limitations in the existing studies [27].

Research indicates that long-term favourable space closure is more likely when early FPMs extraction is performed while the SPMs is in the root development stage E according to Demirjian’s classification. This stage represents to an early phase of root formation, allowing for better spontaneous mesial movement of the SPM [11, 28].

Furthermore, despite theoretical concerns that the absence of the FPM could lead to significant overeruption of the opposing FPM due to the lack of occlusal contact [7], no significant overeruption was observed in this study. Early research suggests that the risk of upper FPM overeruption after the extraction of a lower FPM is relatively low [12, 29]. This is an important clinical finding, as overeruption can complicate future orthodontic treatment [30].

Although, panoramic radiographs are standard imaging method in this type of research study, they can introduce potential measurement errors due to radiographic distortions, despite the calibration and error analysis. A key advantage of this study is that the radiographs were taken at multiple clinics, reducing dependence on a single operator´s imaging technique. The frequency of positioning errors was consistent with findings from previous studies [15].

Since the aim of this study was to study the eruption pattern at 11 years of age, a limitation might be the relatively short follow-up period of about three years, which may not fully capture the long-term consequences of early FPM extractions on the eruption and alignment of SPMs and SPs. However, the study provides valuable insights into the expected outcomes by age 11 following early FPM extraction. To gain a more comprehensive understanding of the full orthodontic implications, future studies should incorporate extended follow-up periods to explore the long-term effects of early FPM extractions in patients with severe MIH.

## Conclusion

Within the limitations of panoramic radiograph measurements, this study found that early extraction of the first permanent molar (FPM) due to severe Molar-Incisor Hypomineralisation (MIH) at age 11 had different effects on eruption patterns in the upper and lower jaws. In the maxilla, the second permanent molar (SPM) and second premolar (SP) erupted perpendicularly, with the SPM erupting faster. However, in the mandible, their eruption patterns were not affected. No overeruption of the opposing FPM was observed. Additionally, no correlation was found between eruption length, angulation, root development, or the presence of a third molar. The study also highlighted errors in panoramic radiographs, mainly due to incorrect patient positioning, such as chin rotation and insufficient tongue contact with the hard palate. These factors may impact measurement reliability.

## Data Availability

The data underlying this article will be shared on reasonable request to the corresponding author.

## Clinicaltrails.gov

Registration number: NCT06228989.

## Permission to reproduce material from other sources

The data are original, and collected by the authors.
